# Activation studies of the α- and β-carbonic anhydrases from the pathogenic bacterium *Vibrio cholerae* with amines and amino acids

**DOI:** 10.1080/14756366.2017.1412316

**Published:** 2017-12-12

**Authors:** Andrea Angeli, Sonia Del Prete, Sameh M. Osman, Fatmah A. S. Alasmary, Zeid AlOthman, William A. Donald, Clemente Capasso, Claudiu T. Supuran

**Affiliations:** a Dipartimento Neurofarba, Sezione di Scienze Farmaceutiche e Nutraceutiche, Università degli Studi di Firenze, Florence, Italy;; b Istituto di Bioscienze e Biorisorse, CNR, Napoli, Italy;; c Department of Chemistry, College of Science, King Saud University, Riyadh, Saudi Arabia;; d School of Chemistry, University of New South Wales, Sydney, Australia

**Keywords:** Carbonic anhydrase, metalloenzymes, pathogens, activators, *Vibrio cholerae*

## Abstract

The α- and β-class carbonic anhydrases (CAs, EC 4.2.1.1) from the pathogenic bacterium *Vibrio cholerae*, VchCAα, and VchCAβ, were investigated for their activation with natural and non-natural amino acids and amines. The most effective VchCAα activators were L-tyrosine, histamine, serotonin, and 4-aminoethyl-morpholine, which had K_A_s in the range of 8.21–12.0 µM. The most effective VchCAβ activators were D-tyrosine, dopamine, serotonin, 2-pyridyl-methylamine, 2-aminoethylpyridine, and 2-aminoethylpiperazine, which had K_A_s in the submicromolar – low micromolar range (0.18–1.37 µM). The two bacterial enzymes had very different activation profiles with these compounds, between each other, and in comparison to the human isoforms hCA I and II. Some amines were selective activators of VchCAβ, including 2-pyridylmethylamine (K_A_ of 180 nm for VchCAβ, and more than 20 µM for VchCAα and hCA I/II). The activation of CAs from bacteria, such as VchCAα/β has not been considered previously for possible biomedical applications. It would be of interest to study in more detail the extent that CA activators are implicated in the virulence and colonisation of the host by such pathogenic bacteria, which for *Vibrio cholerae*, is highly dependent on the bicarbonate concentration and pH in the surrounding tissue.

## 1. Introduction

Carbonic anhydrases (CAs, EC 4.2.1.1) are a superfamily of ubiquitous metalloenzymes with the catalytically active form represented by a metal hydroxide derivative acting as a potent nucleophile on CO_2_ (the physiological substrate) or other electrophiles (e.g. COS, CS_2_, esters, etc.)^1–15^. CAs catalyse only one simple but physiologically highly relevant reaction, which is the reversible hydration of carbon dioxide to bicarbonate and protons^5^^,^^6^^,^^13^^,^^15^. These enzymes are grouped in seven genetically distinct families, named α-, β-, γ-, δ-, ζ-, η- and ɵ-CAs, and although they share a low sequence similarity and protein three dimensional structure, all of them possess a high efficiency as catalysts for the transformation of the metabolically crucial gas CO_2_ into soluble products, HCO_3_^−^ and H^+^ ions^5^^,^^6^^,^^8^^,^^13^^,^^15–17^. As a consequence, these enzymes are ubiquitous in all life kingdoms, being found in Archaea, Bacteria, and Eukaryotes^1–5^^,^^15^. α-CAs are normally present in bacteria and eukaryotes, in which they have been thoroughly investigated^1–5^^,^^15^. In fact many human (h) CAs, of the 15 diverse isoforms known to date, are drug targets for inhibitors acting as diuretics or agents for the treatment of glaucoma, epilepsy, obesity, tumors^16–19^, but recently they started to be considered as possible drug targets for neuropathic pain, cerebral ischemia, or arthritis^20^^,^^21^.

The metal ion from the CA active site is crucial for catalysis, and is coordinated by three His residues in the α-, γ-, δ-, and probably the θ-classes; by one His, and two Cys residues in β- and ζ-CAs or by two His and one Gln residues in the η-class, with the fourth ligand being a water molecule/hydroxide ion acting as nucleophile in the catalytic cycle of the enzyme^1–15^. The rate determining step in the CA catalytic cycle is the formation of the metal hydroxide species of the enzyme from the acidic one in which a water molecule is coordinated as the fourth ligand to the metal centre^3–6^^,^^9^. This process is usually assisted by amino acid residues placed in the middle or at the rim of the active site, which can shuttle protons between the metal centre and the reaction medium by means of moieties possessing a pKa in the region of 6–8 pH units, such as imidazoles (from His residues), carboxylates (from Asp or Glu residues), etc.^3–6^^,^^9^. In α-CAs, the proton shuttle residues are His (e.g. His64 in isoforms, such as CA II, IV, VII, IX, etc.), or His clusters (His3, 4, 10, 15, and 64) placed at the amino terminal part of the protein and situated on the rim of the active site cavity, as demonstrated by X-ray crystal work^3–6^^,^^9^. In β-CAs, which are highly abundant in bacteria and plants, the identity of the proton shuttle residue is not well established although it seems that an Asp (or Glu) residue placed in the middle of the cavity has such a role^22^. Thus, compounds able to intervene in such proton transfer processes are known as CA activators (CAAs) and they were rather well investigated for mammalian α-CAs^23–30^, but much less for bacterial such enzymes. In fact, whereas bacterial CA inhibitors (CAIs) were extensively studied, leading to a detailed understanding of the catalytic and inhibition mechanisms^15^^,^^31–35^, only a few studies are available on the bacterial CAAs^36^. Recently, our groups described the biochemical properties of a α-, β-, and γ-CAs from the pathogenic bacterium *Vibrio cholerae*, responsible of cholera^37–43^. These enzymes, called VchCAα/β/γ showed a significant catalytic activity for the physiologic CO_2_ hydration reaction to bicarbonate and protons (k_cat_ 10^5^ s^−1^)^37–43^. Moreover, the study of the inhibition profiles with the classical CA inhibitors (sulphonamides and anions) revealed interesting structure–activity relationship for the interaction of these enzymes with inhibitors^27–43^, but no activation studies were reported so far. Here, we present the first activation study of two such enzymes, VchCAα/β, with a series of amino acid and amine derivatives. The main interest of this study is to understand whether CA activators are implicated in the virulence and colonisation of the host by this pathogenic bacterium, considering the fact that *V. cholerae* is highly dependent on the bicarbonate concentration and pH in the tissue which is colonised.

## 2. Materials and methods

### 2.1. Materials

Amino acids and amines **1–19** were commercially available, highest purity reagents from Sigma-Aldrich, Milan, Italy.

### 2.2. CA enzyme activation assay

An Sx.18Mv-R Applied Photophysics (Oxford, United Kingdom) stopped-flow instrument has been used to assay the catalytic activity of various CA isozymes for CO_2_ hydration reaction^44^. Phenol red (at a concentration of 0.2 mM) was used as indicator, working at the absorbance maximum of 557 nm, with 10 mM Hepes (pH 7.5) or Tris (pH 8.3) as buffers, 0.1 M Na_2_SO_4_ (for maintaining constant ionic strength), following the CA-catalysed CO_2_ hydration reaction for a period of 10 s at 25 °C. Activity of the α-CA was measured at pH 7.5 whereas that of the β-class enzyme at pH 8.3 in order to avoid the possibility that its active site is closed^40^. The CO_2_ concentrations ranged from 1.7 to 17 mM for the determination of the kinetic parameters and activation constants. For each activator at least six traces of the initial 5–10% of the reaction have been used for determining the initial velocity. The uncatalysed rates were determined in the same manner and subtracted from the total observed rates. Stock solutions of activators (10 mM) were prepared in distilled-deionised water and dilutions up to 1 nM were done thereafter with the assay buffer. Activator and enzyme solutions were pre-incubated together for 15 min (standard assay at room temperature) prior to assay, in order to allow for the formation of the E–A complex. The activation constant (K_A_), defined similarly with the inhibition constant K_I_, can be obtained by considering the classical Michaelis–Menten equation (Equation (1)), which has been fitted by non-linear least squares by using PRISM 3:

(1) $$v\;=\frac{v_{\text{max}}}{\left\{\text{1}+{\text{K}}_{\text{M}}/\left[\text{S}\right]\left(\text{1}+{\left[\text{A}\right]}_{\text{f}}/{\text{K}}_{\text{A}}\right)\right\}}$$

where [A]_f_ is the free concentration of activator.

Working at substrate concentrations considerably lower than K_M_ ([S] **≪**K_M_), and considering that [A]_f_ can be represented in the form of the total concentration of the enzyme ([E]_t_) and activator ([A]_t_), the obtained competitive steady-state equation for determining the activation constant is given by Equation (2):

(2) $$v=\frac{v_{0}•{\text{K}}_{\text{A}}}{\left\{{\text{K}}_{\text{A}}+{\left({\left[\text{A}\right]}_{\text{t}}-0.\text{5}\{\left({\left[\text{A}\right]}_{\text{t}}+{\left[\text{E}\right]}_{\text{t}}+{\text{K}}_{\text{A}}\right)-{\left({\left[\text{A}\right]}_{\text{t}}+{\left[\text{E}\right]}_{\text{t}}+{\text{K}}_{\text{A}}\right)}^{\text{2}}-\text{4}{\left[\text{A}\right]}_{\text{t}}•\;{\left[\text{E}\right]}_{\text{t}}\right)}^{\text{1}/\text{2}}\}\right\}}$$

where *v*_0_ represents the initial velocity of the enzyme-catalysed reaction in the absence of activator^23–30^.

## 3. Results and discussion

**Figure 1.**
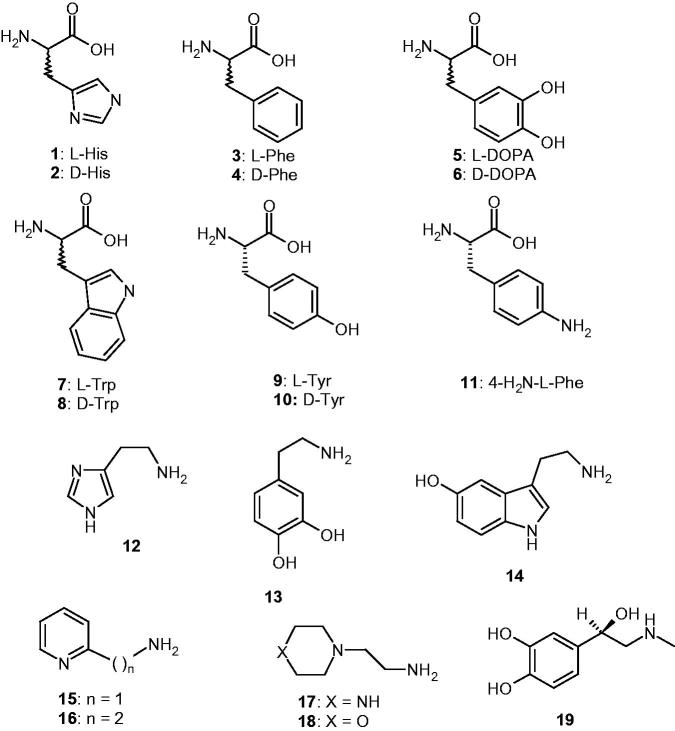
Amino acids **1–11** and amines **12–19** investigated as VchCAα/β activators.

The activators **1–19** were included in this study, as they were employed for investigations as CAAs against many classes of CAs, including the bacterial ones from *Burkholderia pseudomallei*, BpsCAβ/γ^36^^c,^^45^. Both natural and non-natural amino acids and amines were included among the investigated compounds (Figure 1).

Data in Table 1 indicate that L-Tyr (at 10 µM concentration) is an effective activator because this amino acid enhances the k_cat_ values for all enzymes considered (hCA I, II, and VchCAα/β). Moreover, K_M_ remains unchanged by addition of L-Tyr, which has been the case for all CAAs that have been investigated so far, including those belonging to vertebrates (α-class enzymes) and microorganisms (enzymes belonging to various CA genetic families)^23–30^^,^^45^. L-Tyr was a nanomolar activator for the α-class enzymes (hCA I and II) with K_A_s in the range of 11–20 nM^23^ and a micromolar activator for VchCAα/β, with K_A_s of 6.15–8.21 µM. It should be mentioned that due to its high efficacy as activator, L-Tyr induced an increase of the kinetic constant of 2.66 times compared to the uncatalysed rate for the α-CA and of 4.85 times for the β-CA from *V. cholerae*. This is the most significant kinetic effect observed so far any activator that has been identified for these enzymes to date, and L-Tyr is in fact not even the most effective activator of VchCAα/β evidenced here (see below).

**Table 1.** Activation of human carbonic anhydrase (hCA) isozymes I, II, and VchCAα/β with L-Tyr, at 25 °C, for the CO_2_ hydration reaction^44^.

	k_cat_*	K_M_*	(k_cat_)_L-Tyr_**	K_A_*** (μM)
Isozyme	(s^−1^)	(mM)	(s^−1^)	L-Tyr
hCA I^a^	2.0 × 10^5^	4.0	13.9 × 10^5^	0.020
hCA II^a^	1.4 × 10^6^	9.3	12.8 × 10^6^	0.011
VchCAα^b^	8.23 × 10^5^	11.7	21.9 × 10^5^	8.21
VchCAβ^b^	3.34 × 10^5^	8.1	16.2 × 10^5^	6.15

* Observed catalytic rate without activator. K_M_ values in the presence and the absence of activators were the same for the various CAs (data not shown).

** Observed catalytic rate in the presence of 10 μM activator.

*** The activation constant (K_A_) for each enzyme was obtained by fitting the observed catalytic enhancements as a function of the activator concentration^44^. Mean from at least three determinations by a stopped-flow, CO_2_ hydrase method. Standard errors were in the range of 5–10% of the reported values (data not shown).

a Human recombinant isozymes, from Ref^23^.

b Bacterial recombinant enzymes, this work.

Amino acids and amines **1–19** (Figure 1) previously investigated as CAAs of human (α-class CAs) and few bacterial enzymes, showed significant activating effects against VchCAα/β, as observed from data of Table 2, in which the activation constants (K_A_s) of these compounds against four CAs are presented. The following structure-activity relationship (SAR) can be evidenced from the data of Table 2:

**Table 2.** Activation constants of hCA I, hCA II and the bacterial CAs VchCAγ/β with amino acids and amines **1–19**. Data for hCA I and II are from Ref.^23^.

		K_A_ (μM)*			
No.	Compound	hCA I^a^	hCA II^a^	VchCAα^b^	VchCAβ^b^
**1**	L-His	0.03	10.9	43.2	20.3
**2**	D-His	0.09	43	22.7	18.0
**3**	L-Phe	0.07	0.013	53.6	15.4
**4**	D-Phe	86	0.035	34.5	5.12
**5**	L-DOPA	3.1	11.4	23.1	8.36
**6**	D-DOPA	4.9	7.8	19.4	6.27
**7**	L-Trp	44	27	40.9	4.18
**8**	D-Trp	41	12	38.0	5.89
**9**	L-Tyr	0.02	0.011	8.21	6.15
**10**	D-Tyr	0.04^b^	0.013^b^	37.8	0.94
**11**	4-H_2_N-L-Phe	0.24	0.15	41.6	7.21
**12**	Histamine	2.1	125	9.12	9.50
**13**	Dopamine	13.5	9.2	35.2	1.24
**14**	Serotonin	45	50	11.7	1.37
**15**	2-Pyridyl-methylamine	26	34	68.3	0.18
**16**	2–(2-Aminoethyl)pyridine	13	15	71.9	1.00
**17**	1–(2-Aminoethyl)-piperazine	7.4	2.3	57.3	0.24
**18**	4–(2-Aminoethyl)-morpholine	0.14	0.19	12.0	12.8
**19**	L-Adrenaline	0.09	96	18.2	8.73

* Mean from three determinations by a stopped-flow, CO_2_ hydrase method^44^. Standard errors were in the range of 5–10% of the reported values (data not shown).

a Human recombinant isozymes, stopped flow CO_2_ hydrase assay method^25^.

b This work.

(i) The α-class bacterial enzyme was activated by amino acids and amines **1–19** in the micromolar range (K_A_s of 8.21–71.9 µM), and is thus much less sensitive to activation compared to the human CA isoforms belonging to the same class, hCA I and II, because some of these compounds acted as nanomolar activators. However, a distinct SAR could be observed for these CAAs even if their potency is not very high. The most effective VchCAα activators were L-Tyr **9**, histamine **12**, serotonin **14**, and 4-aminoethyl-morpholine **18**, which had K_A_s in the range of 8.21–12.0 µM. The remaining amines and amino acids were less effective CAAs, with K_A_s in the range of 19.4–71.9 µM. The stereochemistry of the amino acid derivatives influenced the activation potency, with the D-enantiomers being generally more effective than the L-ones (for His, Phe, DOPA, and Trp), whereas the reverse situation is true for Tyr, case in which the L-enantiomer was 4.6 times more effective at activation than the D-enantiomer (Table 2). In some cases, the amines were more effective activators compared to the amino acids structurally related to them, e.g. histamine was more effective compared to L/D-His, whereas dopamine was less effective compared to L/D-DOPA. The least effective activators were the pyridyl-amine derivatives **15** and **16**. All these data demonstrate that relatively small differences in the scaffold of the activator induce important differences in the activation efficacy, obviously due to the fact that the structural diversity of these compounds induces diverse interactions with amino acid residues from the active site in the enzyme-activator (E-A) complex.

(ii) VchCAβ was more sensitive to activation with the amines and amino acids investigated here, which showed K_A_s in the range of 0.18–20.3 µM (Table 2). The most effective activators were D-Tyr **10**, dopamine **13**, serotonin **14**, 2-pyridyl-methylamine **15**, 2-aminoethylpyridine **16,** and 2-aminoethylpiperazine **17**, which showed activation constants in the submicromolar – low micromolar range, of 0.18–1.37 µM. Apart D-Tyr, all of these most effective activators are amines. Another subset of derivatives, such as **4–9, 11, 12, 18**, and **19** were slightly less effective CAAs with K_A_s in the range of 4.18–12.8 µM. They include both amino acid and amine derivatives. The least effective activators were L/D-His and L-Phe, with K_A_s in the range of 15.4–20.3 µM. Again, generally D-enantiomers of the amino acids were generally more effective activators compared to the L-enantiomers (for His, Phe, DOPA, and Tyr), whereas in the case of Trp, the L-enantiomer was a better activator compared to the D one (Table 1).

(iii) There are important differences in activation efficacy of these amino acids and amines against the two bacterial enzymes, with the β-class one being much more sensitive to activation compared to the α-class. There are also important differences of the activation profiles of these compounds for the bacterial and human CAs, which is a rather important observation as this may lead to isoform-selective activators. However, for this small panel of activators, the human CAs were generally much better activated compared to the bacterial enzymes, with few exceptions, such as the activity of **13–17** for VchCAβ which was much more susceptible to be activated compared to hCA I, II, and VchCAα. This observation demonstrates that it may be possible to design bacterial CA – selective activators.

## 4. Conclusions

The first activation study of two CAs from the bacterial pathogen *Vibrio cholerae* is reported here, with a series of amino acid and amine derivatives. The most effective VchCAα activators were L-tyrosine, histamine, serotonin, and 4-aminoethyl-morpholine, which had K_A_s in the range of 8.21–12.0 µM. The most effective VchCAβ activators were D-tyrosine, dopamine, serotonin, 2-pyridyl-methylamine, 2-aminoethylpyridine, and 2-aminoethylpiperazine, which showed activation constants in the submicromolar – low micromolar range, K_A_s of 0.18–1.37 µM. The two bacterial enzymes had very different activation profiles with these compounds, between them, and also when compared to the human isoforms hCA I and II. Some amines were VchCAβ – selective activators. The activation of CAs from bacteria, such as VchCAα/β, was never considered up until now for possible biomedical applications. It would be of interest to study in more detail whether CA activators may contribute to processes connected with the virulence and colonisation of the host by such pathogenic bacteria, which as *Vibrio cholerae*, is highly dependent on the bicarbonate concentration in the tissue.
